# Supplementary material for: Are carbon emissions trading and green financial instruments synergistic? -Comprehensive quantitative research based on content analysis
Source: PLoS One. 2024 Mar 7;19(3):e0298601. doi: 10.1371/journal.pone.0298601 (PMC10919601; doi:10.1371/journal.pone.0298601)
Supplement: S1 Data — (ZIP) [file pone.0298601.s001.zip › Data/地级市Z_F工作报告.zip!/╝¬┴╓/╝¬┴╓╩╨/└┤╫╘╣1⁄2╓┌║┼í╛┬φ┐╦╩2╛▌═°í┐_2017.docx]

各位代表：

　　现在，我代表市政府向大会报告工作，请予审议，并请政协委员提出意见。

　　一、本届政府和2016年工作回顾

　　自十五届人大一次会议以来，我们在省委、省政府和市委的正确领导下，在市人大、市政协的监督支持下，团结带领全市人民，努力克服经济下行压力加大、困难矛盾集中显现等诸多严峻挑战，主动适应新常态，统筹稳增长、调结构、促改革、惠民生，克难攻坚，砥砺前行，经济社会发展取得重大成就，圆满完成了本届政府任期工作任务。

　　经济质量持续提升，综合实力显著增强。推进经济结构战略性调整，三次产业比重调整到10.2：44.3：45.5，服务业比重高出全省6个百分点，现代农业建设走在全省前列，“6411”产业体系架构基本形成。民营经济对经济总量贡献率突破50%、地方税收贡献率达到76.8%。实施亿元以上重点项目1073项，固定资产投资累计完成9738亿元，年均增长14.3%。全市地区生产总值年均增长6.9%，人均生产总值达到5.9万元。全口径财政收入占GDP比重达到12.5%。

　　城市功能日趋完备，城乡面貌明显改善。累计完成城建投资985亿元，建成12座桥梁、78条主次干路，新建改建各级各类公路1621公里，“八纵八横”城市路网初步形成；组织实施节能减排、低碳城市等一批国家试点示范，环境保护和治理成效显著，绿化、美化、亮化水平大幅提升，生态宜居、休闲度假的城市功能品质充分彰显。县域发展能力持续增强，特色鲜明的县城新形象进一步展现，小城镇和新农村建设取得新成效，城镇化率达到59.1%。

　　改革开放成果丰硕，发展活力有效释放。稳妥推进重点领域改革，一些制约发展的深层次矛盾得到有效破解，行政体制、市场体系改革富有成效，国资国企、社会体制、民主法制改革取得阶段性成果。对外开放开创新局面，重点功能区开放带动作用和产业集聚效应凸显，万丰工业园、海王健康产业基地等一批重大招商项目落地建设，累计招商引资到位资金3503亿元，实际利用外资39.6亿美元。

　　人民生活稳步提高，社会事业全面进步。坚持每年将新增财力的80%用于保障和改善民生，累计投入423亿元，一大批民生事项和惠民工程高质量完成，城镇和农村常住居民人均可支配收入年均分别增长7.1%和7%。城乡就业稳步扩大，累计新增城镇就业38万人；社会保障体系日趋完善，城乡养老保险制度在全省率先实现全覆盖；基础教育、职业教育保持省内领先，公共卫生保障能力大幅提升，文化事业蓬勃发展，竞技体育实现奥运金牌“零突破”。社会安全稳定局面持续巩固。第八次荣获“全国双拥模范城”称号。

　　党的群众路线教育实践活动、“三严三实”专题教育和“两学一做”学习教育深入开展，政府执行力、工作作风和整体形象发生了积极变化。

　　各位代表，2016年是“十三五”开局之年。面对复杂多变的经济形势，我们紧紧围绕建设旅游文化名城、新型产业基地、生态宜居城市，从重点指标、重点项目、重点工程、重点工作四个方面精准发力，主动作为，较好地完成了年度185项重点任务，经济社会保持稳中有进、稳中向好的发展态势。预计实现地区生产总值2530亿元，增长7%；规模工业增加值751亿元，增长6%；固定资产投资2871亿元，增长11.2%；社会消费品零售总额1438亿元，增长9.5%；地方级财政收入135.5亿元，增长2%；城镇常住居民人均可支配收入25305元，农村常住居民人均可支配收入12300元，分别增长6%和7%。　　——稳增长取得新成效。力促工业平稳运行。把稳工业作为核心任务，实施一系列精准调控措施。加大“上争”力度，为31户企业争取各类专项及奖补资金4亿元。深入开展银企保对接，净增工业贷款35亿元，为中小企业担保贷款100亿元。全力组织减亏增盈，45户“双停”企业恢复生产，亏损企业亏损额下降35%；百户增量企业新增产值100亿元，重点建成项目达产达效率超过70%；规模工业利润实现110亿元，增长85%。强化投资拉动。集中开展征地拆迁、融资、招商、手续办理等系列攻坚活动，实施5000万元以上重点项目684项，其中亿元以上415项、10亿元以上48项；高密度组织多轮务实招商活动，新开工5000万元以上招商项目300项，招商引资到位资金1200亿元，增长12%。着力扩大消费。优化消费环境，培育消费热点，健康养老、家政服务、医疗保健等新兴消费需求加速释放，华业国际广场、百业国际五金汽配城一期等一批提升业态水平的大型商业综合体竣工运营，首届中国吉林市·新加坡商贸周活动取得丰硕成果。电子商务发展势头强劲，“一网全城”等区域电商品牌效应放大，吉林乌拉电商产业园、筑石电商产业园成立运营，舒兰“村淘产地仓”建成，棋盘、黄松甸等“淘宝村”建设全面启动，全市网络零售额增长56.8%。

　　——调结构迈出新步伐。新兴产业发展态势良好。突出“一江一带”旅游发展核心，万科松花湖旅游度假区、温泉带综合开发、朱雀山国家森林公园等景区景点建设大力推进，四季精品旅游线路不断完善，以冰雪游、温泉游为代表的旅游市场持续升温，旅游总收入增长25.4%。医药健康产业快速成长，长白山制药、吉尔吉药业等骨干医药企业保持较快发展，优势产品体系不断健全，产值增速高于全市规模工业10个百分点。云计算产业中心初具规模，大仝软件外包基地建成使用，培育国家“两化”融合试点企业10户，占全省的37%。着力发展通航经济，二台子机场改扩建审批工作取得阶段性成果，山河多用途无人机实现量产，福航航空学院开展学员培训工作。碳纤维产业战略发展地位凸显，纳入全省高位统筹，与中国复合材料集团成功签订战略合作协议，吉研高科复合材料等一批产业链项目实施，吉林碳谷200吨大丝束碳化示范线建成投产。恒涛节能锅炉、旭峰科技3D打印等先进装备制造业项目稳步推动。传统产业改造步伐加快。推进化工“补链”，吉神化工30万吨环氧丙烷产能释放，康乃尔60万吨甲醇制烯烃顺利实施，吉化汽油国V质量升级改造竣工投产。推进汽车“上量”，一汽大众经济型轿车项目积极推进，一汽吉林森雅R7车型产销超4万辆，T80双排座微卡具备年产2万辆生产能力，吉星轮胎、龙山减速器等汽车零部件项目实现量产。推进冶金“转型”，吉林铁合金、吉林炭素签订搬迁框架协议，12万吨碳素及制品等技改升级项目加快实施。推进农产品加工“提质”，新建续建31个重点项目，新晋3户国家级龙头企业，被认定为国家级出口食品农产品质量安全示范区。现代服务业水平不断提升。安中危化品、博宇医药、晟驰汽车等一批物流园区集聚发展效应增强，市保税物流中心通过国家验收。金融体系日趋完善，招商银行、盛京银行、长春农商行在我市设立分支机构，村镇银行实现地区全覆盖，昌邑金融集聚区获批筹建，金融业增加值增长19.6%。房地产市场逐步回暖，商品住房去库存周期同比下降3.9个月。现代农业体系建设扎实推进。万昌、孤店子现代农业先导区引领作用提升，全国农业结构调整现场会在我市召开。优质稻米品牌创建成果明显，蔬菜、中药材等50个标准化园区建设加快，新建、改扩建标准化养殖场215个，长白山特产业规模效应显现。智慧农业成为发展新亮点，云耕智慧农业平台投入使用，建成23家可视农业基地。金珠花海、二道采摘、旺起湿地、东福稻乡等休闲农业发展方兴未艾，休闲农业星级企业发展到75户，数量居全省第一。

　　——强动力实现新突破。重点领域改革不断深化。较好完成供给侧结构性改革5大方面25项年度任务。市属经营性国有资产完成划转并集中统一监管，市场化选聘国企经理层成员试点展开，混合所有制改革进行了有益探索。“五证合一”“一照一码”“先照后证”登记制度全面实施，企业注册实现全程电子化，市场主体同比增长11.9%。民用天然气、供热、交通、旅游等相关价格改革取得阶段性成果。投融资改革收到实效，组建产业发展基金10支、规模超百亿元，吉歌传媒等12户企业在“新三板”挂牌，温德河湿地公园等14个PPP合作项目纳入财政部项目信息平台。户籍制度和居住证制度改革扎实推进，现代学徒制试点稳步开展，公立医院和基层医疗卫生机构综合改革不断深化，医保支付制度改革实现全覆盖。国有林场改革试点、供销合作社综合改革进展顺利，农村土地确权提前一年完成三年实测任务，发放农村土地经营权抵押贷款、收益保证贷款3.8亿元，被列为国家农村金融改革试点地区。创新驱动战略深入实施。推进产学研用深度合作，特种纤维等一批细分行业应用技术研究院成立，东北首家国家科技领军人才创新驱动中心建立，市级以上企业技术中心达到99个，100项科技攻关及产业化项目加速实施，科技“小巨人”企业发展到206户。推动大众创业、万众创新，巨邦软件园等载体建设加快，创新科技园投入运营，培育众创空间、创业孵化基地各10个；发放创业担保贷款1.8亿元，扶持创业企业2215户；成功举办“互联吉林、创赢江城”创新创业大赛，有效激发了全社会的创新创业活力。

　　——惠民生收获新成果。脱贫攻坚成效显著。大力开展精准脱贫春季和秋季攻势，落实包保帮扶、专家把脉、产业造血等系列措施，筹集脱贫资金3.5亿元，落实脱贫产业项目816个，68个村、2.8万户、4.9万人脱贫出列，完成贫困村、贫困户“双60%”脱贫任务。社会保障体系进一步完善。机关事业及企业退休人员养老金月人均分别增长6%和7%，工伤、失业、生育保险费率降低，长期护理保险制度试点启动，城乡低保提标全面落实。居民生活条件有效改善。继续开展“温暖家庭、整洁楼道、和谐社区、畅通街路”创建活动，完成134条街巷、47条胡同和53个老旧小区综合改造任务。完成棚户区改造7266套，回迁安置7150户。改造“暖房子”36.2万平方米，解决近7万户居民房证办理问题。维修改造给排水、供热、燃气等各类管网306公里。新开、调整公交线路29条，新增公交车150台。新建改建各级各类公路558公里。建设城区水洗公厕69座、改造农村厕所1.05万户。大力开展农村“六清”整治行动，村容村貌进一步改观。启动50个贫困村安全饮水工程，解决5.4万农村居民饮水安全问题。生态环境进一步优化。全面开展“长吉平”三市共治大气污染专项行动，空气质量优良天数超出上年48天。实施最严格水资源管理制度，持续推进松花江流域综合整治，水环境质量持续改善。清收还林成果显著，还林54.8万亩，森林覆盖率达到54.9%。社会事业全面发展。教育“阳光行动”深入推进，完善优质资源共享区，组建城区大学区88个；取得全国职业学校技能大赛历史最好成绩。加强医疗卫生三级网络建设，分级诊疗制度稳步推进，242个村级“一站式”服务平台建成，公共卫生医疗服务水平进一步提升。国家公共文化服务体系示范区创建进展顺利，冰雪奥运冠军培育计划大力实施，广场文化周、全民上冰雪等文体活动蓬勃开展；高水平举办吉林市首届国际马拉松赛、首届中国道教文化艺术周等重大节庆赛事，城市的迷人魅力充分彰显，知名度和影响力明显扩大。

　　——法治政府建设开创新局面。“放管服”改革扎实推进，取消调整行政审批事项86项，规范清理行政审批中介服务128项，权责清单监管机制进一步健全。启动政务服务“一张网”建设，政务云平台投入使用，部分行政审批、公共服务事项上线运行。全过程电子化公共资源交易平台体系初步建立，在全省率先开展县（市）限额以下公共资源交易。重大行政决策咨询委员会组建运行，为市政府科学决策提供了强有力的智力支撑。

　　各位代表，过去几年，吉林市走过了不平凡的发展历程，经济社会发展跃上新的台阶，为在全省率先全面建成小康社会奠定了坚实基础。这些成绩的取得，是市委科学决策、坚强领导的结果，是市人大、市政协加强监督、鼎力支持的结果，是全市上下众志成城、拼搏进取的结果。在此，我代表市政府，向全市450万人民，向辛勤工作在各条战线的广大建设者，向各级人大代表、政协委员、各民主党派、工商联和社会各界人士，向中省直单位、驻吉部队指战员和武警官兵，向始终关心支持吉林市发展的海内外朋友，表示衷心的感谢和崇高的敬意！

　　我们也清醒地认识到，发展中还存在不少困难和问题：传统产业转型艰难，新兴产业竞争力不强，新旧动能接续转换还需较长一段过程；自主创新能力较弱，创新创业氛围不浓，内生动力活力尚需进一步释放；公共服务保障能力不足，一些关系人民群众切身利益的问题还没有很好解决，改善民生任务依然艰巨而繁重；政府依法行政水平有待提高，政务服务亟待加强，发展环境需要进一步改善。对于上述问题，我们一定高度重视，采取有效措施认真加以解决。

　　二、新一届政府和2017年工作安排

　　今后五年，是全面建成小康社会的决胜阶段和城市转型发展的关键时期。刚刚闭幕的市第十三次党代会在综合研判外部形势的基础上，科学确定了今后一个时期我市的发展思路和目标任务。我们将按照市第十三次党代会和“十三五”规划部署，坚持稳中求进工作总基调，牢固树立和贯彻落实新发展理念，适应把握引领经济发展新常态，紧紧围绕省委“三个五”发展战略和“六个吉林”建设，加快打造旅游文化名城、新型产业基地、生态宜居城市，着力推进“四化”联动，着力强化改革创新，着力提高开放水平，着力建设法治政府，在全省率先全面建成小康社会，奋力开创老工业基地振兴发展的新局面。

　　按照“三翻番、三超越、三提升”的目标要求，未来五年发展预期目标是：地区生产总值年均增长7%，规模工业增加值年均增长5.5%，三次产业比重调整到7：41：52，固定资产投资年均增长9%，社会消费品零售总额年均增长9%，地方级财政收入年均增长5%，城镇、农村常住居民人均可支配收入与经济增长同步，生态环境指标位于全省前列。

　　今后五年，我们要在发展中牢牢把握好以下几点：

　　——坚持一张蓝图绘到底。把建设旅游文化名城、新型产业基地、生态宜居城市作为我市转型发展的战略引领，咬住既定目标不放松，持续发力，久久为功。围绕建设旅游文化名城，突出“一江一带”旅游发展核心，深入挖掘和彰显特色文化内涵，推进旅游与文化、体育、健康、休闲等关联产业深度融合，打造独具魅力的国家全域旅游示范区和全国特色文化城市。围绕建设新型产业基地，紧扣转型升级主线，坚持继承和创新并重，加快建设“6411”现代产业体系，建成国家老工业基地转型升级示范区和新兴特色产业集聚区。围绕建设生态宜居城市，优化城市空间布局，巩固山水生态优势，完备综合服务功能，着力构筑生态良好、特色鲜明、生活舒适的品质江城。

　　——坚持率先发展不动摇。加大重点方面和关键环节的突破力度，切实担负起在全省率先全面建成小康社会的职责和使命。以新一轮振兴发展为核心，加快经济结构战略性调整，推进服务业、民营经济、县域经济发展质量效益实现新超越，率先完成经济总量翻番目标。以脱贫攻坚为突破口，加大民生保障和改善力度，率先完成城乡居民收入翻番目标。以深化“放管服”改革为重点，下大力气破除体制机制障碍，率先全面建成法治政府。

　　——坚持集中力量办大事。从战略上牵住发展的“牛鼻子”， 集中人力、物力、财力，持续建设一批牵动力强、影响深远的重大项目，切实发挥项目建设对振兴老工业基地的支撑作用。围绕城市发展定位，滚动实施千亿级产业培育计划、百亿级项目（工程）实施计划，加速形成新的经济增长点。积极构建市场化合作、多渠道投入、滚动式开发的项目运作模式，建立健全重大项目目标责任、协调服务和督查考评工作机制。牢牢抓住“一带一路”、新一轮东北振兴等战略机遇，谋划储备一批打基础、利长远的重大项目，切实增强经济社会发展后劲。

　　2017年，是新一届政府开局之年。主要预期目标是：地区生产总值增长7%左右，规模工业增加值增长6.5%，固定资产投资增长8%左右，社会消费品零售总额增长9.5%，地方级财政收入增长3%（结合经济企稳向好趋势，按前低后高安排），城镇和农村常住居民人均可支配收入与经济增长同步，节能减排完成省下达任务。　　（一）实施五大升级工程，加快建设旅游文化名城

　　以创建全域旅游示范区为统领，推进旅游文化产业提档升级，旅游文化业增加值占GDP比重15%以上。

　　实施冰雪产业升级工程。抓住我国举办2022北京冬奥会的有利契机，以“雪”为主，开发双顶山、莲花山雪上运动核心圈，启动北大壶国家青少年雪上训练基地二期、冬奥村和奥体中心建设，争取国家级冰雪训练基地落户我市；抓好乌拉街曾通和渔楼、小白山乡青山、舒兰二合等最美关东雪乡建设，完善雾凇岛景区配套设施，建设雾凇室内体验馆。以“冰”为主，开发东山冰上运动核心圈，启动建设速滑馆、冰球馆和校园滑冰馆等一批冰上基础设施；引入专业冰球队，彰显城市品牌形象。积极承办国际雪联空中技巧世界杯、世界雪日暨国际儿童滑雪节等大型节庆赛事，举办万人上冰雪及冰雪雕塑、冰雪摄影等冬令文化活动100场。

　　实施松花湖景区升级工程。加快松花湖国家5A级旅游景区创建工作，构建环湖旅游经济圈。完善松光景区、湖光景区基础设施，实施环湖旅游公路及7条景区连接线改扩建工程，建成景区水上交通指挥中心等配套设施；连片开发松花湖景区，推进卧龙潭、北天门“龙珠湾”、双峰岭、北雪秘境乐园综合开发，启动朱雀阁、丰满风情小镇建设，打造旺起大自然生态区。开展近湖区生态恢复及综合治理，整合50万亩渔业资源，做大做强松花湖渔业产业。推出朱雀山“满韵清风”大型驻场演出，高水平举办开江鱼美食节、休闲避暑度假节、红叶旅游节、冬捕冬钓等四季特色活动，打响松花湖旅游品牌。

　　实施温泉产业升级工程。编制完成温泉产业发展规划，系统提升万昌——孤店子温泉产业带建设水平。加大扶持和配套建设力度，推进搜登站、桦皮厂等7个温泉休闲节点镇建设，快速形成产业优势和集群效应。创新温泉+医疗、+养生、+养老、+农业等发展模式，重点实施欧乐堡极地海洋冰雪世界、圣德泉温泉旅游养生园、白桦林家温泉、祥康养老中心、长吉养老社区等温泉产业项目，建设一批旅居集聚区，打造一批健康旅游产品。

　　实施文化产业升级工程。建设玄天岭文化公园、乌拉古城、苏密古城等项目，启动广电传媒中心建设。打造国家级影视基地，组织拍摄《红灯记》《光影江城》等影视作品。举办国际国内演艺娱乐活动，精心打造玫瑰音乐节等文化品牌，组织艺术演出200场以上。依托文化艺术中心、美术馆等平台，组织开展国家级书画艺术写生与交流活动；高水平、市场化运营第二届国际马拉松赛、中华龙舟赛等赛事，进一步扩大我市对外知名度和影响力。

　　实施城市品牌升级工程。创新营销渠道，加强整体策划，高水平、系统化推介“中国雾凇之都”“中国冰雪名城”“中国最美温泉带”“天下第一江山”等城市品牌。大力开展创建全国文明城行动，广泛发动，全民参与，打好公共环境、公共服务等专项整治战役，深化文明单位、文明社区、文明村镇、文明校园、文明家庭创建活动，全面展示城市文明发展的新形象。补齐公共服务短板，完善重点景区给排水、垃圾转运等设施，智慧旅游平台、游客服务中心投入运行，开通连接火车站与万科滑雪场、神农温泉等旅游专线，实现3A级以上景区、二级以上公路互联互通。

　　（二）构建现代产业体系，加快建设新型产业基地

　　以提高质量和核心竞争力为中心，切实抓好“6411”产业体系建设，着力振兴实体经济。

　　突出高端引领，做强新兴产业。新材料产业：整合资源，筹建产业集团，推进碳纤维产业化、规模化发展。深化与中建材、中复材合作，开工建设吉林碳谷4万吨碳纤维原丝等项目，启动搬迁方大1500吨碳化生产线，吉林精功1.2万吨大丝束碳化一期投产。航空产业：推进二台子机场改扩建工程，实施瀚星格莱赛尔飞机组装制造等重点项目，吉航民机维修项目建成投产，争创国家通用航空产业综合示范区。健康产业：整合药号资源，做大优势品种，打造以维生素E、咖啡因为重点的原料药生产基地，以北沙制药、北京四环医药为支撑的医药中间体生产基地；规划建设长白山医药产业园、修正健康产业园。信息产业：完善通信基础设施，4G基站比例提升到50%；加快中国联通全省云数据基地、华微新型电力电子器件基地建设，培育“两化”融合试点企业20户以上。先进装备制造产业：推进特种机器人生产基地建设，开工圆方机械高性能大型农机驱动桥、森远智能跨座式单轨除冰机等项目，恒涛节能锅炉等达产达效。

　　紧扣转型升级，改造传统产业。围绕化工“补链”，延伸基础化工产业链，做好吉化40万吨ABS项目前期工作；延伸生物化工产业链，推进中能环科60万吨秸秆资源化综合利用项目建设，博大生化20万吨无水乙醇项目建成投产；延伸资源综合利用产业链，实施大地化工硫磷钛综合利用项目二期，瀚宇化工3.6万吨二萘酚项目建成投产；延伸丙烯及聚氨酯产业链，规划建设聚氨酯产业园，开工康乃尔能量优化及富余氢气资源综合利用项目，推动60万吨甲醇制烯烃项目一期建成投产。围绕汽车“上量”，推动一汽吉林森雅R7车型月产超万辆，龙山微型卡车、面包车生产能力达5万辆；启动马来西亚宝腾年产20万辆整车建设，全力推进一汽大众DY系列A0级SUV落地；实施万丰工业园二期、凯德传动轴、恒达金型等汽车配套项目。围绕冶金“转型”，加快哈达湾老工业区搬迁改造，吉林铁合金、吉林炭素力争完成整体搬迁，同步启动新厂区建设；支持建龙钢铁转型升级、扩大融资，推进年产80万吨冷轧钢项目。围绕农产品加工“提质”，实施广泽奶酪加工等19项农业产业化重点项目，市级以上龙头企业发展到290户，农产品加工销售收入增长12%。支持中新食品区争创中国食品安全国际合作试验区，打造特色农产品出口基地。

　　坚持需求导向，提升现代服务业。突出发展电子商务。立足建设东北电子商务中心城市，深化与阿里巴巴战略合作，重点抓好吉林乌拉电商产业园、5个县域电子商务服务中心、4个“淘宝村”建设；增加社区电商服务网点布局，完善“一站式”便民服务功能。全市网络零售额同比增长35%以上。改造提升传统商贸业。以河南街、东市场等老商圈改造为重点，引入智慧商圈设计理念，实现线上线下融合发展，推动传统商贸焕发新活力。积极发展现代物流业。加快长吉图物流园、“一网全城”电商物流园等重点项目建设，提升重点物流园区集聚能力；抓好保税物流中心封关运营，尽快发挥农产品进出口交易中心、跨境贸易融资服务中心等核心功能。加快发展现代金融业。引导银行业金融机构完成信贷投放1000亿元以上；启动金融总部基地建设，大力发展昌邑金融集聚区；培育10户企业上市挂牌；推动东北亚国际商品交易中心投入运营；成立金融服务公司，弥补企业贷款担保短板。促进房地产业平稳健康发展。科学优化开发增量，全面实施不动产登记，加大回迁安置力度，积极培育发展房屋租赁市场。

　　建设三大体系，发展现代农业。以农业供给侧结构性改革为引领，促进农业增效、农民增收，争当现代农业发展排头兵。推进产业体系建设。优化种植业结构，调减玉米种植面积1.5万公顷以上。深入实施“健康米”工程，着力发展设施农业，推动果蔬、食用菌等十大优势特色产业形成绿色品牌优势，规划建设温德河现代都市农业产业带。启动实施万亩森林中药材、万亩绿化苗木等8大林业产业工程，促进林下经济发展。抓好畜牧业标准化、规模化养殖，提高粮食过腹转化增值水平，全域无疫区建设力争一次性通过国家评估验收。推进生产体系建设。抓好万昌、孤店子现代农业先导区建设，打造现代农业示范基地；实施农业物联网示范工程，信息进村入户覆盖率达到50%；建设323项农田水利改造提升工程；完成肉菜流通追溯体系试点建设，国家区域食品农产品检验检测中心投入使用。推进经营体系建设。有序流转农村土地12万公顷以上，农业专业合作社、家庭农场分别发展到4400户和2000户，新认证“三品一标”农产品25个。

　　（三）完备综合服务功能，加快建设生态宜居城市

　　坚持绿色、共享和可持续的发展理念，加快建设品质江城。

　　巩固生态环境优势。围绕创建生态文明先行示范区，坚持保护和治理并重，推动生态环境建设再上新台阶。实施蓝天工程。深入开展“长吉平”三市共治大气污染专项行动，提升生物质能源综合利用水平，积极引进秸秆开发利用新技术、新项目，加快取缔小锅炉，空气质量优良天数保持270天以上。实施碧水工程。推进松花江流域综合整治，启动温德河沿岸整体开发，温德河湿地公园投入使用；整治远大河、落马湖等九河一湖黑臭水体，切实改善松花江干流水质，集中式饮用水源水质保持三类标准。实施沃土工程。建立全市土壤信息数据库，推动国家级矿山生态恢复治理项目落地，启动一批企业厂区土壤环境治理项目；持续开展农药化肥零增长行动；巩固清收还林成果，再还林30万亩以上。实施宜居工程。以深入开展“温暖家庭、整洁楼道、和谐社区、畅通街路”创建活动为引领，全面提升城市美化、绿化、亮化、硬化、净化水准，新增城市绿地42公顷，新建改建水洗厕所50座，完成77个老旧小区宜居改造和114条街巷整治任务；继续加大“六清”投入，深入开展农村人居环境提升工程，启动乡镇垃圾集中无害化处理试点建设，改造农村厕所2.5万户。

　　加强城市建设管理。实施城建重点工程103项，完成投资130亿元。新建续建白山大桥等4座桥梁，开工滨河景观路等62条街路，建设松花江两岸百里慢行系统。加快微管廊建设，改造架空线缆，实施供水应急备用水源管线、六水厂、“一城一网”供热管网联网工程，完成23项路桥维修改造任务。升级改造各级各类公路787公里，吉哈高速吉林至荒岗段主线贯通。搭建智慧化城市管理平台，规范道路停车和停车位管理，加强弃管小区物业管理，强化违法建筑、野广告、散流体等专项整治，全面构建城市网格化管理新格局。

　　切实保障改善民生。坚持尽力而为、量力而行，努力增进民生福祉。保基本。城镇新增就业9万人，农村富余劳动力转移就业65万人；城市、农村低保标准分别提高到每人每月513元和每人每年3660元；推进社会养老保险征缴扩面，确保及时足额发放；整合并统一城镇居民医保和新农合医保，逐步实现跨省异地医疗费用直接结算；加强保障房分配管理，为7500户城镇低收入住房困难家庭发放租赁补贴，集中分配公共租赁住房2108套。兜底线。继续开展脱贫攻坚行动，深入落实产业扶贫、专项扶贫等政策措施，解决所有贫困村安全饮水问题，全面完成贫困村和贫困人口“双脱贫”任务。补短板。实施学校教师招聘面试改革、名优教师培养建设工程，调整优化城区学校布局，新建改建公办幼儿园17所、改造农村薄弱校22所。启动市科技馆建设，打造“互联网+”少年儿童科普体验平台。全面推开各级各类公立医院改革，健全分级诊疗制度，实施儿童医院、妇产医院改扩建等项目，不断提高医疗卫生和计划生育服务水平。完善公共文化服务网络，全面做好国家公共文化服务体系示范区创建工作；创新经营管理模式，探索成立广电集团。大力实施冰雪奥运冠军培育计划，推动冰雪体育运动进校园，全年实现学生上冰雪10万人次。促和谐。严格落实安全生产责任制，持续开展安全生产隐患排查整改和食品药品安全示范创建行动，坚决杜绝重特大安全生产事故发生。进一步加大“事要解决”力度，妥善处理好人民群众的合理诉求。推进平安吉林建设，严厉打击非法集资，全力维护社会和谐稳定。开展好“七五”普法。做好双拥优抚安置工作，促进军地融合发展。

　　为完成上述目标任务，我们将着力夯实三大支撑：

　　（一）强化投资拉动，夯实转型发展的项目支撑

　　年内组织实施5000万元以上项目700项，其中亿元以上项目450项。

　　发挥重大项目工程牵引作用。重点实施超百亿元重大产业项目10项，重大城镇化工程10项，预计总投资3106亿元，年度完成投资423亿元。

　　十大产业项目：即计划投资380亿元的航空产业园、投资283亿元的冰雪体育运动核心圈、投资215亿元的吉林科技创新城、投资204亿元的化工产业“补链”、投资122亿元的哈达湾老工业区搬迁改造、投资117亿元的“一江三湖”旅游开发、投资114亿元的温泉产业带综合开发、投资108亿元的汽车产业“上量”、投资102亿元的碳纤维产业化基地、投资101亿元的健康医药产业基地。

　　十大城镇化工程：即投资222亿元的中新食品区产城融合、投资155亿元的城市地下综合管廊、投资151亿元的文化景观基础设施、投资141亿元的城市轨道交通、投资130亿元的长春路区域城市改造与更新、投资126亿元的松花江百里生态长廊、投资114亿元的公路交通重点工程、投资110亿元的温德河流域生态治理及开发、投资106亿元的城市棚户区改造、投资105亿元的城市快速路。

　　推进投融资机制创新。整合成立百亿级产业投资引导基金，搭建覆盖重点产业的子基金体系，精准投向重点项目和企业。引进优质融资中介资源，发展多渠道直接融资，实现低成本融资200亿元以上。加快建设投资项目数据平台，大力引入银行、保险公司、投资机构，探索实施融资议价、竞价制度，实现项目金融需求线上实时对接和最优匹配，构建“互联网+项目+金融”的快速通道。

　　完善重大项目推进机制。健全目标责任机制，做到“一个重大项目（工程）、一位市级包保领导、一个责任主体、一个融资计划、一套实施方案、一揽子抓到底”。健全协调服务机制，强化全程跟踪、主动对接，着力协调解决项目招商、开工、在建、竣工、投产各个环节的突出问题；强化市、县两级征迁批办领导小组职能，着力解决房屋土地征收和手续办理问题。落实招商引资主体责任，制定招商引资政策和季度招商计划，强化专业招商、产业招商、平台招商和委托招商，努力实现招商引资的新突破，年内新开工重点招商项目200项、到位资金1350亿元。健全督查考评机制，制定可量化、可操作的评价标准，采取月调度、季巡检、年考评方式，推动重大项目建设不断取得新成效。

　　（二）强化改革创新，夯实转型发展的动力支撑

　　以供给侧结构性改革为主线，以创新发展为引领，充分激发经济社会发展的内生动力。

　　深化重点领域改革。推进国资国企改革。创新运营模式，组建文投集团，壮大四大平台公司发展实力，加快资源资产化、资产资本化、资本证券化进程；实施企业分类改革，推行市场化公开选聘高级管理人员制度，全面提升国企经营管理水平；推动驻吉央企“三供一业”分离移交等专项改革，争取市属国有企业列入首批混合所有制改革试点。推进市场体系改革。探索开展售电侧改革，组建售电公司，争取吉林化纤自备电站获批建设，促进企业用电成本大幅降低；推进水、天然气、交通、供热、旅游、公共医疗等领域价格改革，放开竞争性环节价格，健全市场决定价格机制。推进财税体制改革。加强结转结余资金管理，着力盘活财政存量资金；完成三年中期财政规划编制，强化规划对年度预算的约束；国库集中支付及授权支付电子化业务上线运行。推进农村综合改革。完成农村土地确权登记颁证工作，开展农村集体产权制度改革试点；加快供销社、集体林权改革步伐，全面完成国有林场改革任务。

　　实施创新驱动战略。大力推进科技创新。加快科技创新城建设步伐，完成10所中职学校搬迁入驻职教园区并顺畅运行，启动2所高职学院搬迁新建，推动职教园区与中德工业园、医药健康园、创新科技园等同步建设、融合发展，打造科技创新集聚区；深化产学研用协同创新，创建化学原料药、特种纤维等产业创新联盟，引进中科院应化所中试基地、光机所创新孵化器和百度创新中心，推进英联生物等研发平台建设，实施新产品、新技术、新工艺开发项目700项，实现新产品规模化生产100种。深入开展“双创”工作。设立10亿元科技创业基金，支持智慧农业产业园、蚁神动漫等创业孵化园区发展，引导风险投资、创业投资、天使投资等资本投向“双创”领域，推动众创、众包、众扶、众筹等新业态发展；提升“双创”服务平台功能，支持初创企业1000户，完成创业培训1万人，发放创业担保贷款1亿元；完善扶持政策，突出抓好百户“专精特新”企业培育发展，全年新增“四上”企业205户以上，民营经济主营业务收入增长7.5%以上。实施“高层次紧缺人才引进计划” “蓝领人才培育计划”，继续举办创新创业大赛，加速集聚“双创”人才队伍；发挥驻吉高校人才、科研等优势，建立联席会议制度，推动政、校、企深度对接，全面加快校城融合发展步伐。

　　（三）强化自身建设，夯实转型发展的政务支撑

　　以“放管服”改革为统领，以从严从实作风为保障，加快转变政府职能，努力建设人民满意的法治政府。

　　加大“放”的力度。进一步增强放权协同性，承接落实好国家和省下放事权，动态调整市级行政审批事项目录，凡是目录外事项一律不得审批，凡是新增目录事项一律严控，凡是基层能够办理的事项一律下放。进一步深化商事制度改革，推动“五证合一”“一照一码”广泛应用，推广工商网上登记平台，放宽企业名称登记限制，扩大简易注销登记试点范围，努力解决企业“准入不准营”问题。进一步清理规范中介服务，尽快公布行政审批中介服务事项目录，切断各类利益关联，破除各种服务垄断，切实为企业和群众松绑。

　　做好“管”的文章。健全清单管理机制，严格依照权力清单、责任清单和公共事项服务清单履职用权，加快建立企业投资项目管理负面清单，完善“四张清单”动态调整机制，坚决做到法定职责必须为、法无授权不可为。推行“双随机一公开”，加快建立随机抽查事项清单、检查对象名录和执法检查人员名录，细化抽查程序，实行联合监管和综合执法，坚决避免重复检查，切实减轻企业负担。完善社会信用体系，建立以居民身份证、组织机构代码证为基础的统一社会代码制度和市场主体诚信档案，升级改造企业信用信息公示系统平台，完善联合惩戒信用监管机制，全面营造公平、公开、公正的市场环境。

　　提升“服”的质量。围绕建设新型智慧城市，重点抓好政务服务“一张网”，构筑以五大平台为支撑的“互联网+政务”新型服务体系。建设政务云平台，归集构建人口信息、法人单位、地理空间、宏观经济四大基础数据库，逐步实现各级各部门业务数据互联互通。建设网上行政审批平台，开发网上并联审批系统和移动客户端发布系统，建立实体大厅和网上大厅有机结合的行政审批新机制。建设网上公共服务平台，实现公共服务事项网上公开、网上咨询申报和办理、网上支付、网上评价和投诉。完善网上政府信息公开平台，有序拓展行政决策、执行、管理、服务和结果“五公开”范围，深度推行阳光政务。完善网上公共资源交易平台，理顺公共资源交易管理体制和运行机制，建立全市一体化运行体系，实现公共资源交易全程电子化。

　　强化“严”的作风。坚持严字当头、实处用力，把作风建设成效转化为推动发展的强大动力。严明政治纪律，牢固树立政治意识、大局意识、核心意识、看齐意识，坚决贯彻省委、省政府和市委的决策部署，自觉接受市人大、市政协及社会各界监督。严格决策程序，按照决策事项目录，认真履行公众参与、专家论证、风险评估、合法性审查、集体讨论决定的法定程序，充分发挥市决咨委委员智囊作用，有效提高政府决策科学化水平。严肃绩效考评，实行重点工作目标责任制，强化责任担当，强化督查问效，强化考核问责，以“钉钉子”精神持续提升政府执行力。严厉整风肃纪，加强软环境建设，加大涉软案件查处力度，坚决整治行政不作为、乱作为等问题，努力打造一流的营商环境；加强政府系统党风廉政建设，从严落实“一岗双责”，从严惩治腐败问题，切实做到干部清正、政府清廉。

　　各位代表！使命在肩，唯有奋发作为；蓝图绘就，更需百倍努力。让我们在省委、省政府和市委的坚强领导下，坚持创新引领、实干担当，加快建设旅游文化名城、新型产业基地、生态宜居城市，以经济社会发展的新成绩迎接党的十九大胜利召开！
